# TLR4-Mediated Placental Pathology and Pregnancy Outcome in Experimental Malaria

**DOI:** 10.1038/s41598-017-08299-x

**Published:** 2017-08-17

**Authors:** Renato Barboza, Flávia Afonso Lima, Aramys Silva Reis, Oscar Javier Murillo, Erika Paula Machado Peixoto, Carla Letícia Bandeira, Wesley Luzetti Fotoran, Luis Roberto Sardinha, Gerhard Wunderlich, Estela Bevilacqua, Maria Regina D’Império Lima, José Maria Alvarez, Fabio Trindade Maranhão Costa, Lígia Antunes Gonçalves, Sabrina Epiphanio, Claudio Romero Farias Marinho

**Affiliations:** 1 Departamento de Ciências Biológicas, Universidade Federal de São Paulo, Diadema, Brazil; 2 Departamento de Parasitologia, Instituto de Ciências Biomédicas, Universidade de São Paulo, Sao Paulo, Brazil; 3 Hospital Israelita Albert Einstein, São Paulo, Brazil; 4 Departamento de Biologia Celular e do Desenvolvimento, Instituto de Ciências Biomédicas, Universidade de São Paulo, São Paulo, Brazil; 5 Departamento de Genética, Evolução e Bioagentes, Instituto de Biologia, Universidade Estadual de Campinas, Campinas, Brazil; 6 Departamento de Imunologia, Instituto de Ciências Biomédicas, Universidade de São Paulo, São Paulo, Brazil; 7 Departamento de Análises Clínicas e Toxicológicas, Faculdade de Ciências Farmacêuticas, Universidade de São Paulo, São Paulo, Brazil

## Abstract

Malaria-associate pregnancy has a significant impact on infant morbidity and mortality. The detrimental effects of malaria infection during pregnancy have been shown to correlate with immune activation in the placental tissue. Herein we sought to evaluate the effect of Toll-like receptors (TLRs) activation on placental malaria (PM) development by using the *Plasmodium berghei* NK65^GFP^ infection model. We observed that activation of the innate immune system by parasites leads to PM due to local inflammation. We identified TLR4 activation as the main pathway involved in the inflammatory process in the placental tissue since the absence of functional TLR4 in mice leads to a decrease in the pro-inflammatory responses, which resulted in an improved pregnancy outcome. Additionally, a similar result was obtained when infected pregnant mice were treated with IAXO-101, a TLR4/CD14 blocker. Together, this study illustrates the importance of TLR4 signalling for the generation of the severe inflammatory response involved in PM pathogenesis. Therefore, our results implicate that TLR4 blockage could be a potential candidate for therapeutic interventions to reduce malaria-induced pathology both in the mother and the fetus.

## Introduction

Gestational malaria has an important impact on infant morbidity and mortality. While it often leads to intrauterine complications, prenatal exposure to *Plasmodium* parasites can shorten the time between birth and a first clinical malaria episode and increase it frequency in the first two years of life^1^. The complications caused by *Plasmodium* infection during pregnancy such as spontaneous abortion, intrauterine growth retardation and low weight at birth^2,3^ are related to the accumulation of infected red blood cells (iRBCs) in the placenta^2,4,5^. The placental tissue creates a maternal-fetal interface that plays an important immunological function as a barrier against microbial infection, recognizing and responding to pathogens via innate pattern recognition receptors (PRR) and thus, preventing injuries in the fetal development^6,7^. Previously, we demonstrated that the adverse placental alterations observed in Plasmodium-infected pregnant mice correlated with MyD88 expression, a key component of the Toll-like receptor (TLR) signalling pathway^8^.

It has been shown that distinct receptors of the TLR family recognize components of the *Plasmodium* parasite, such as the glycosylphosphatidylinositol (GPI) and the hemozoin-DNA complex, stimulating an immune response. Previous reports have evidenced that GPI is recognized both by TLR2 and TLR4^9^, whereas hemozoin, a metabolite derived from the parasite heme detoxification, has been implicated in TLR9 stimulation^10–13^. Several adaptor proteins, such as MyD88, Tirap (also known as Mal), Trif and Tram, mediate TLR activation, which culminates in a pro-inflammatory signalling^14^. Franklin and collaborators^15^ disclosed that, in acute malaria, the TLR9 association with MyD88 is essential for the initiation of an IL-12/IFN-γ-mediated immune response, and the subsequent increased expression of pro-inflammatory cytokines.

In pregnant women, TLR polymorphisms have been associated with an increase in the susceptibility to placental malaria^16–18^. Although several studies have indicated that TLR2, TLR4, and TLR9 are pivotal for the host immune responses to *Plasmodium* infection, and are associated to pathogen-associated molecular patterns^10,11,14,15^, the role of these TLRs in *Plasmodium* infection during pregnancy is not completely elucidated. Even more, the key mechanism for the immune activation following *Plasmodium* infection during pregnancy is not well understood. Using a mouse model for placental malaria infection, our study aimed to evaluate the detrimental effects of the severe inflammatory response via TLR4 signalling in the pathogenesis of malaria during pregnancy. Also, we wanted to assess if treatment of infected pregnant mice with a TLR4 blocker can reverse the placental damage induced by the malarial infection.

## Results

### Experimental malaria infection during pregnancy induces a mononuclear cell infiltration in the placenta

**Figure 1:**
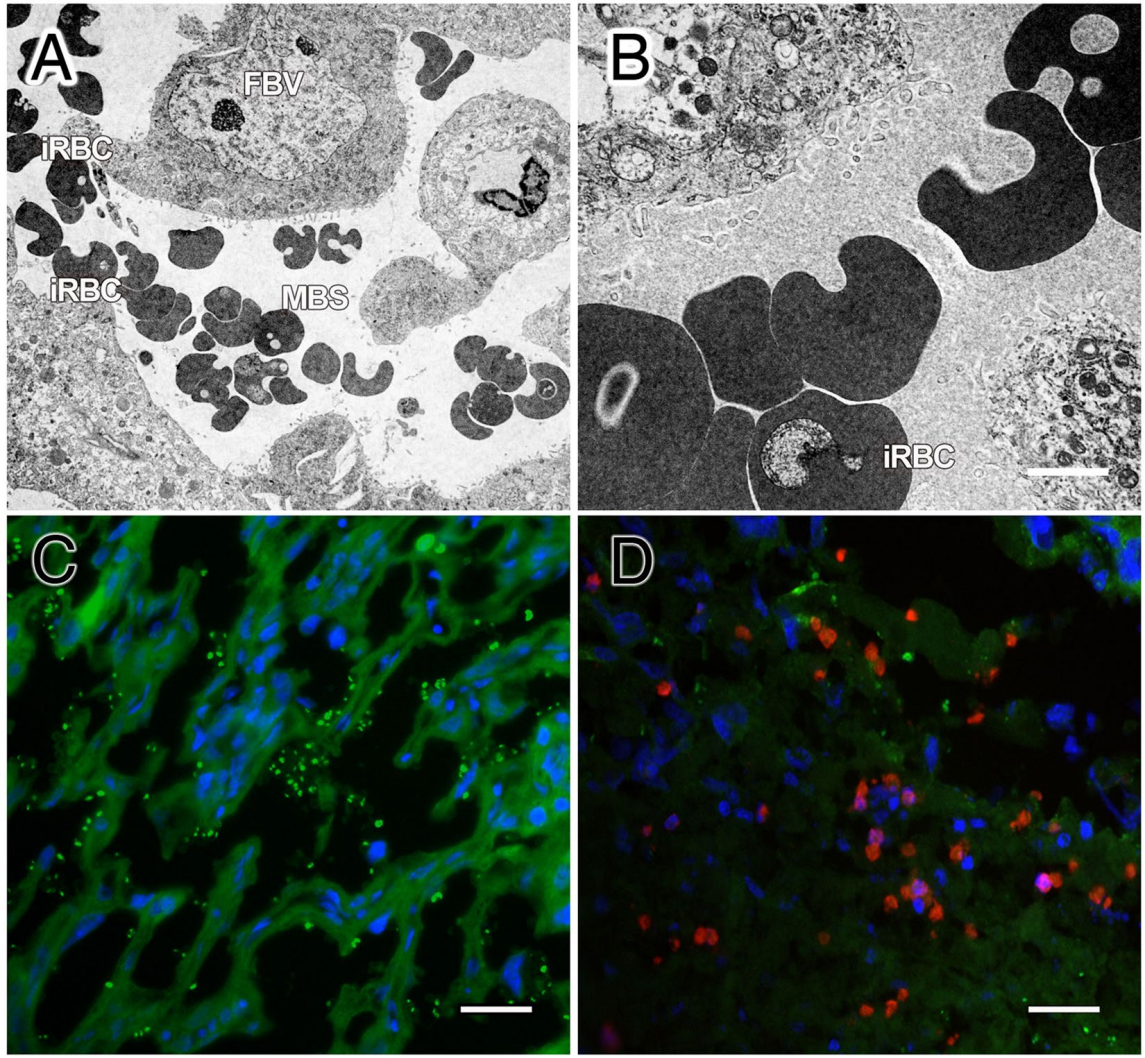
Inflammatory cell infiltration in *P*. *berghei* infected placenta. Images from the placental labyrinth layer from WT infected mice. Transmission electron microscopy (TEM) in the labyrinth region (**A**,**B**) showing the maternal-fetal interface with presence of iRBCs in the maternal blood space (MBS), but not in fetal blood vessels (FBV). Bar in (A) = 10 µm and (B) = 2.5 µm. Immunofluorescence of placentas (**C**,**D**) from infected WT mice with GFP-labelled *P*. *berghei* NK65^GFP^ (green), DAPI (blue) and anti-CD11b (red) revealing the accumulation of parasites (**C**) and infiltration of monocytes/macrophages (**D**) on maternal blood sinuses; Bar in (C,D) = 100 µm. (GC) Giant cell.

Placental tissue is critical for mother-fetuses exchange of nutrients. Any abnormalities in this tissue have an enormous impact on fetal growth and development. To evaluate the effects of malaria infection at the maternal-fetal interface, we first examined the ultrastructure of labyrinth layer from infected mice placentas. As shown in Fig. 1A and B, iRBC were only found in the maternal blood space (Fig. 1A and B). The immunofluorescence staining of maternal blood sinuses from the labyrinth shows an accumulation of GFP-labelled iRBC (Fig. 1C) and mononuclear cells (CD11b^+^ cells). Gene expression analysis of monocytes/macrophages (CD68), dendritic cells/macrophages (Mgl2), neutrophils (Ncf2) shows an increase of these cells in placental tissue from infected mice when compared with the non-infected ones. While the analysis of T and NK cell did not present significate differences (Supplementary Fig. S1). These observations suggest that malaria infection induces an inflammatory cell infiltration composed predominantly of monocytes/macrophages (Fig. 1D).

### *P*. *berghei* infection stimulates TLRs expression in the placenta

**Figure 2:**
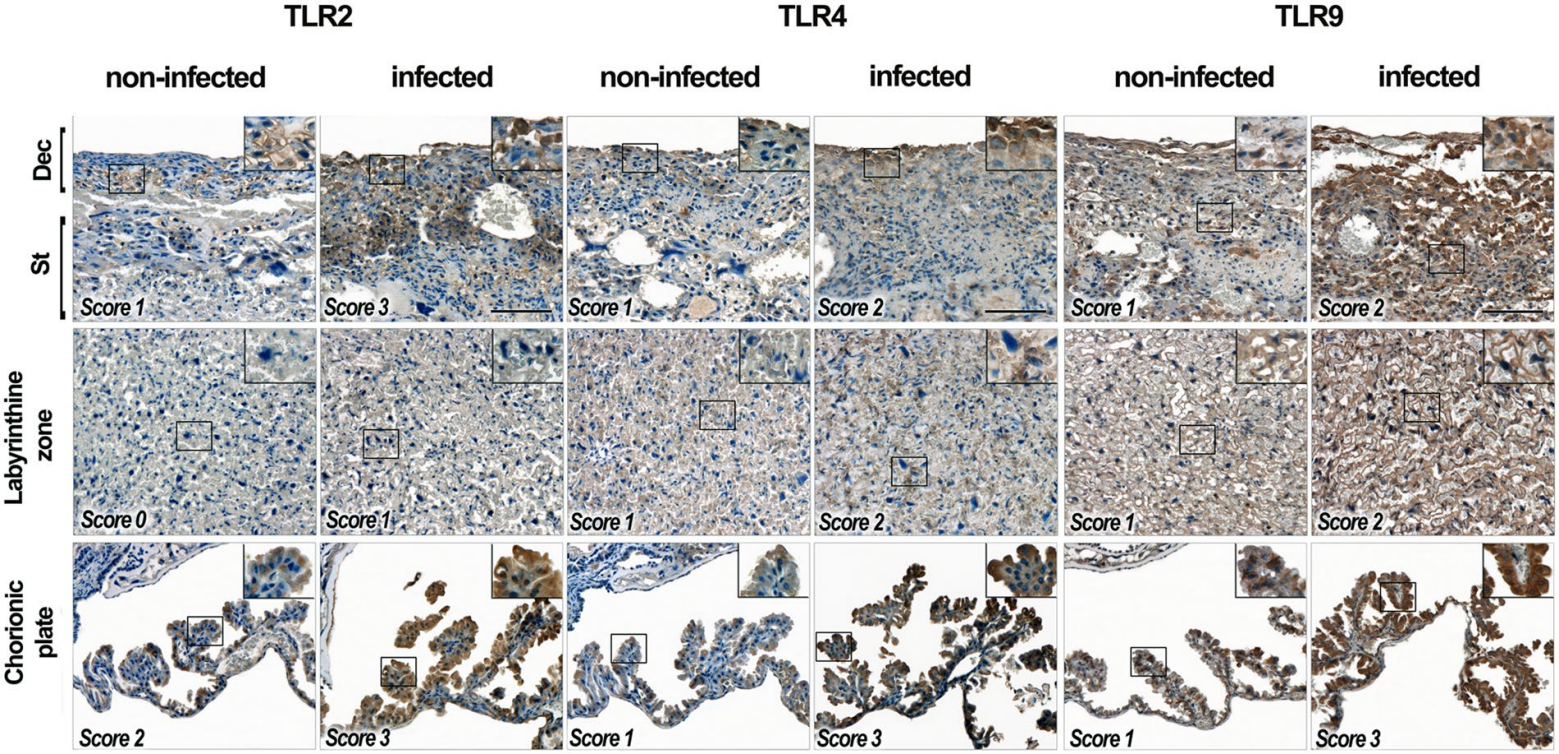
TLRs immunohistochemistry. TLR2, TLR4, and TLR9 immunolabeling in placentas of non-infected and infected mice. Pregnant mice were i.v. infected or not with 1 × 10^5^ *P*. *berghei* NK65^GFP^ iRBCs on the 13^th^ gestational day. Scores of TLR2, TLR4, and TLR9 expression were defined by using protein expression level of positively stained cells (absent = 0, faint = 1, moderate = 2, intense = 3). We considered as “low expression” TLR intensity scores of 0, 1 or 2, and as “high expression” scores of 3. ×200 magnification, ×400 inset. Bar = 100 μm. Dec = decidua; St = Spongiotrophoblast.

To evaluate TLR genes expression in the placental tissue, we conducted a semi-quantitative immunohistochemical analysis of TLR2, TLR4, and TLR9 in different placental regions of infected and uninfected mice: chorionic plate and labyrinth; spongiotrophoblast and decidual layers. As shown in Fig. 2, the chorionic membrane and the decidual region of infected mice presented higher expression of all monitored TLRs in comparison with those from non-infected mice. Decidua and syncytiotrophoblast in the labyrinth region presented lesser expression of TLR2 and TLR4 compared to TLR9 in both groups of mice. Except for the chorionic membrane of the control group, TLR9 was highly expressed in all regions of both placental groups. Gene expression analysis of TLR2, TLR4 and TLR9 on the entire placental tissue, shows an increase of TLR4 and TLR9 in placental from infected mice when compared with non-infected ones (Supplementary Fig. S2). However, in contrast with the immunohistochemical analysis of distinct placental regions, when we analyse the entire placental tissue we do not observe differences in the expression of TLR2 gene of both groups. These results indicate that *P*. *berghei* infection induces an increase of TLRs expression in murine placenta when compared with non-infected groups.

### Placental vascular space impairment is associated with TLR4 activation

Next, we sought to determine if iRBCs with *P*. *berghei* NK65^GFP^ could activate the TLR2, 4 and 9. This was done by COS-7 cells transiently transfected with TLR2, TLR4, and TLR9, and NF-κB-dependent E-selectin promoter luciferase reporter construct. Ours results show that E-selectin reporter expression was increased upon stimulation with iRBCs of cells transfected with TLR2, TLR4 or TLR9. This demonstrates that the parasite used in our model can activate these receptors (Supplementary Fig. S3).

**Figure 3:**
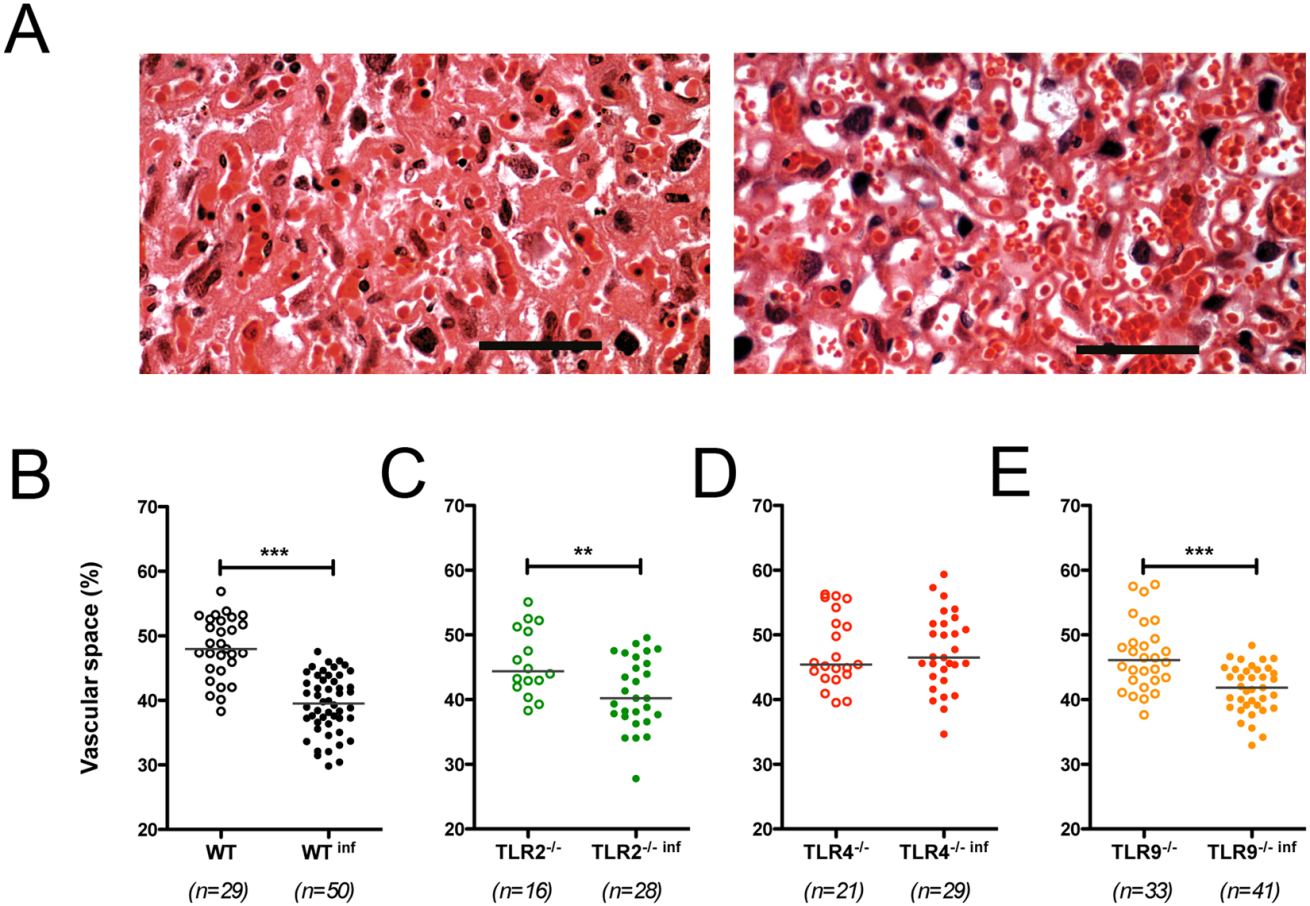
Decreased vascular placental space is associated with the expression of TLR4. Pregnant knockout and WT females were i.v. infected with 1 × 10^5^ *P*. *berghei* NK65^GFP^ iRBCs on the 13^th^ gestational day. Photomicrography of sinusoidal areas of the placenta showing a reduced area in the case of infected placentas (**A**, right) in comparison with non-infected placentas (**A**, left) on G19. Sinusoidal areas of the placenta were quantified in relation to the total area of the placenta by using an automated morphometric analysis method from infected and non-infected WT (**B**), TLR2^−/−^ (**C**), TLR4^−/−^ (**D**), TLR9^−/−^ (**E**), mice. Data presented as scatter plot with an indication of the median of percent vascular placental space area. Unpaired t-test: **P-value < 0.01; ***P-value < 0.001. 100 μm Scale bar.

Using a morphometric method to quantify the area of blood sinuses cross-sections of the placentas, as previously reported^2,8,19^, we observed a reduction in the labyrinth blood sinusoids area in the WT-infected pregnant mice compared with non-infected pregnant WT mice (48.13 ± 0.88% vs. 39.44 ± 0.64% of maternal blood space in non-infected and infected mice, respectively; P < 0.001, mean ± SE) (Fig. 3A and B). We also analysed the blood sinusoid area in the TLR2^−/−^, TLR4^−/−^ and TLR9^−/−^ pregnant infected and non-infected mice (Fig. 3C–E, respectively). Similar to the WT-infected mice, the infected TLR2^−/−^ and TLR9^−/−^ mice displayed a reduction in the blood sinusoid area compared with the non-infected mice (Fig. 3C and E) while TLR4^−/−^ infected mice did not exhibit such a decrease in the blood sinusoid area (Fig. 3D). When we examined the blood sinusoid area of the infected groups, we observe a significate difference only between WT and TLR4^−/−^ infected mice.

In order to assessed the influence of the TLRs on the susceptibility to malaria, we compared the parasitemia of infected pregnant mice on the 6^th^ day post-infection, paired with non-pregnancy controls. The 6^th^ day was chosen to assess parasitemia, since females were euthanized at this point in all experiments. As shown in Supplementary Fig. S4, both TLR2^−/−^ and TLR9^−/−^ infected pregnant mice were more susceptible to malarial infection than non-pregnant controls, while TLR4^−/−^ mice did not show differences between the groups (pregnant and non-pregnant).

### Placental pathology is associated with TNF-α gene expression induced by TLR4 activation

**Figure 4:**
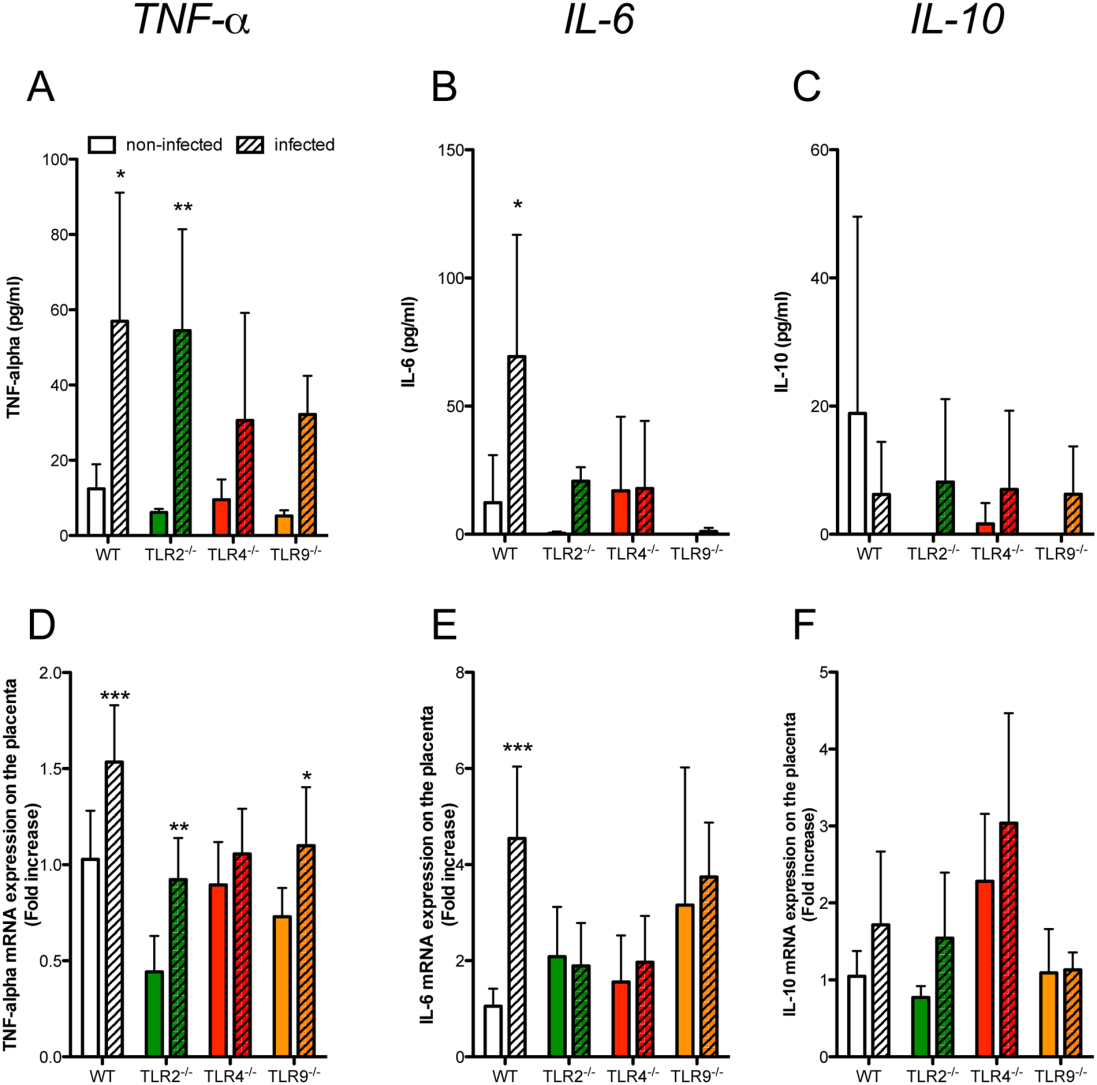
Placental pathology is associated with altered TNF-α gene expression. Serum levels of TNF-α (**A**), IL-6 (**B**) and IL-10 (**C**) measured by cytometric bead arrays (CBA) from WT, TLR2^−/−^, TLR4^−/−^, TLR9^−/−^ mice infected (hatched bars) or not infected (clear bars) with *P*. *berghei* NK65^GFP^. Placental gene expression was quantified for *Tnf* (**D**), *Il6* (**E**) and *Il10* (**F**). mRNA expression was measured in placentas from WT, TLR2^−/−^, TLR4^−/−^, TLR9^−/−^ not infected (clear bars) or infected (hatched bars) mice with *P*. *berghei* NK65^GFP^. Samples were obtained at G19 (6^th^ day post-infection). Differences in steady state mRNA levels were calculated by comparison with the internal control GAPDH as described. The results were plotted as the fold-change over non-infected WT mice and each bar represents the mean ± SE (5 placentas/group from different donors). Two-way ANOVA: *P-value < 0.05; **P-value < 0.01; ***P-value < 0.001.

Since malaria infection did not cause changes in the placental tissue in TLR4^−/−^ mice, we hypothesized that the infection leads to an inflammatory state, which is dependent on the TLR4 activation. Therefore, our next step was to investigate the inflammatory profile associated with the *P*. *berghei* NK65^GFP^ infection in pregnant mice. The analysis of the production of the TNF-α, IL-6 and IL-10 cytokines in the serum demonstrated an increase of TNF-α production in WT and TLR2^−/−^ mice while only a visible but non-statistically significant increase in TNF-α production in TLR4^−/−^ and TLR9^−/−^ all infected mice compared to non-infected was detected (Fig. 4A). The production of IL-6 was higher only in the infected WT mice (Fig. 4B) and no significant differences in the IL-10 serum levels were recorded (Fig. 4C). Except for IL-6, we do not observe a difference in the production of these cytokines between WT and TLR knockout infected groups.

Next, we assessed the effects of the parasite infection on the pro-inflammatory markers gene expression in placental tissue (Fig. 4D–F). Except for TLR4^−/−^ mice, an increase in TNF-α expression in all infected mice was observed when compared with non-infected controls (Fig. 4D). In addition, the placental IL-6 expression increased only in the WT-infected mice (Fig. 4E). No differences were observed regarding the IL-10 among the groups (Fig. 4F). In contrast with the protein production, the TNF-α expression in WT infected mice was higher than TLR knockout infected groups. Regarding the expression of IL-6, WT infected mice shows higher levels of mRNA only when compared with TLR2^−/−^ and TLR4^−/−^, while no difference was observed in the expression of IL-10.

### Reduced growth rate in progeny of infected mothers is correlated with TLR4 expression

**Figure 5:**
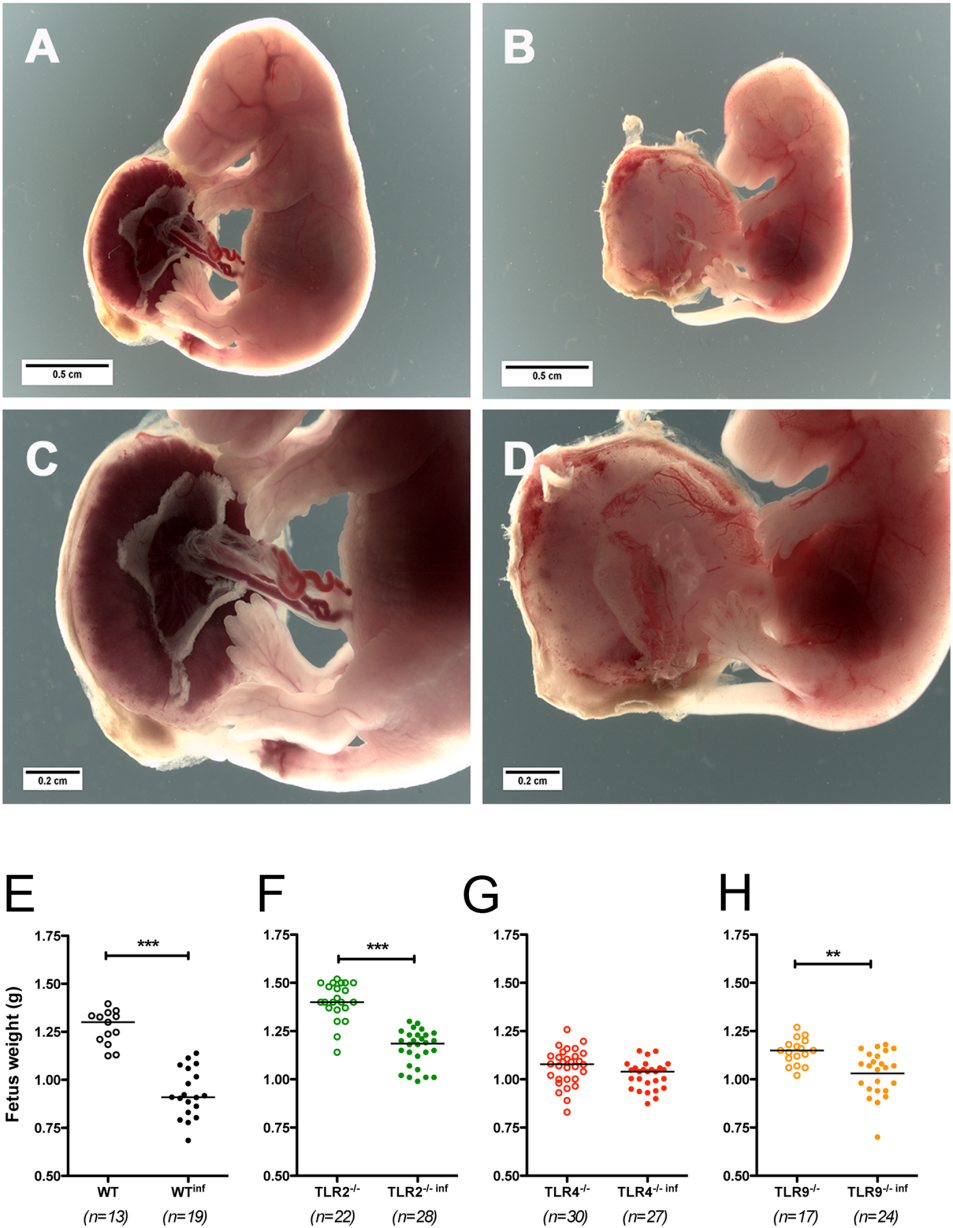
Reduced growth rate in the progeny of infected mothers’ correlates with the expression of TLR4. Pregnant females were infected on G13 by i.v. injection of 1 × 10^5^ iRBCs. Photomicrographs of fetuses and placentas from non-infected (**A**,**C**) and infected (**B**,**D**) WT mothers. Fetal body weight from WT, TLR2^−/−^, TLR4^−/−^, TLR9^−/−^ non-infected and infected mice at G19 (**E**–**H**). Data are presented as scatter plots with an indication of the median. Unpaired t-test: **P-value < 0.01; ***P-value < 0.001.

Placental inflammation seems to provoke impairment of the progeny growth rate. We observed a huge reduction in placental blood irrigation in fetuses and placentas from infected mothers when compared with the controls (Fig. 5A–D). To assess the impact of the placental inflammation on the fetus, its weight was analysed at G19. Consistent with previous studies^2^, fetuses from the WT-infected mothers presented a 30% reduction in body weight compared to those from non-infected WT mice (Fig. 5E). Similar results were observed in fetuses from TLR2^−/−^ and TLR9^−/−^ mothers (Fig. 5F and H, respectively). However, fetuses from TLR4^−/−^ infected mothers did not exhibit differences in body weights when compared to those from non-infected mothers (Fig. 5G). It is important to note that the mouse strains used herein present significant difference in their fetuses’ body weight. This makes impossible any kind of comparative analysis between the strains.

MyD88 and TRIF mediate TLR4 activation^20^. We have previously shown that MyD88 expression is associated with a decreased growth rate^8^. Accordingly, we verified the effect of the TRIF and MyD88 on the body weight of fetuses from infected mice carrying knockouts in these factors. As a result, only fetuses from infected WT mice presented a reduction in body weight while neither fetus from TRIF^−/−^ nor from MyD88^−/−^ infected mice presented differences in their body weight when compared with the controls (Supplementary Fig. S5).

**Figure 6:**
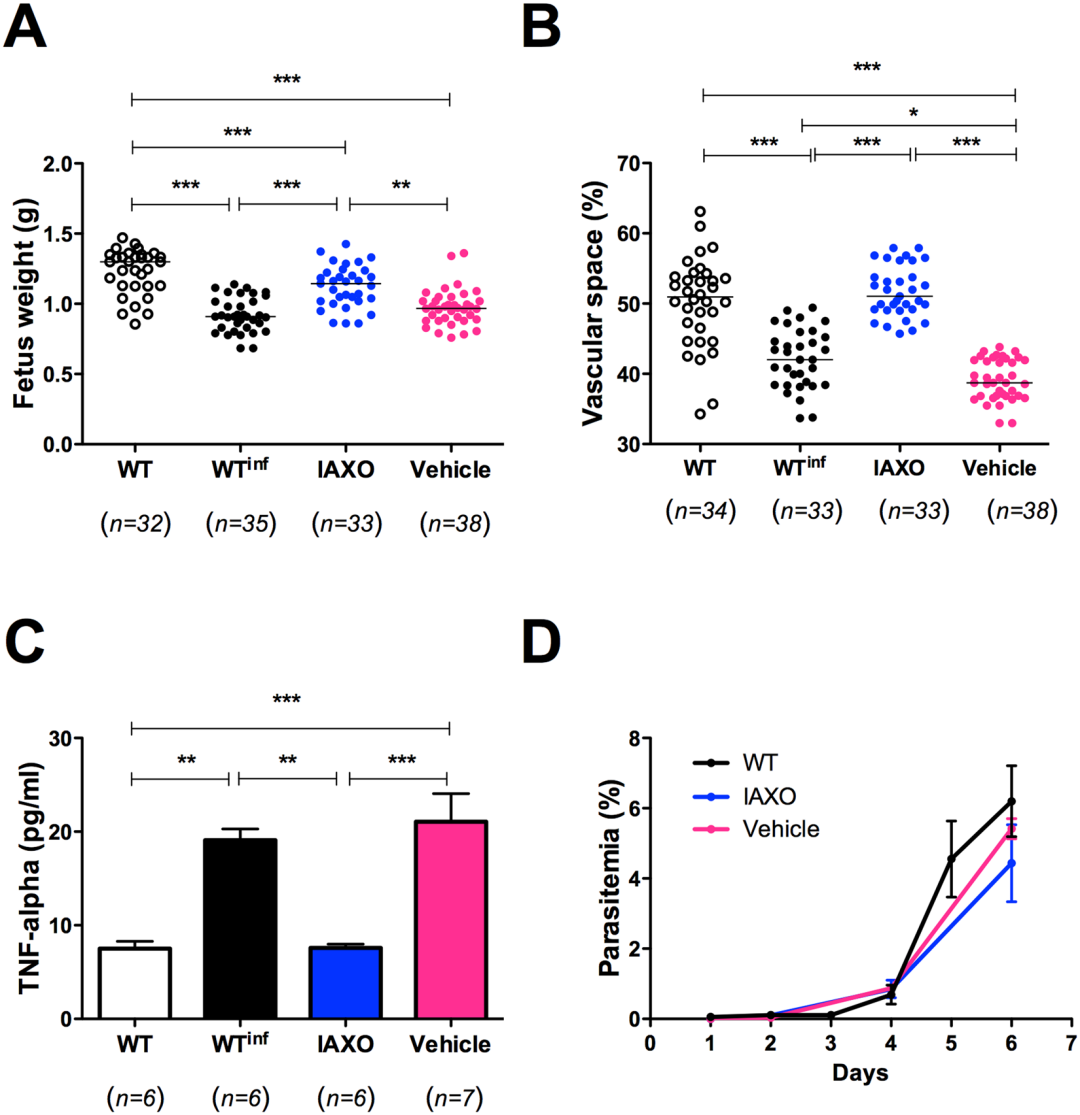
Partial inhibition of the effect of malaria in the fetus development of pregnant C57BL/6 mice infected with *P*. *berghei* NK65^GFP^ by using IAXO-101 as TLR4 antagonist. Fetuses weight from pregnant WT mice infected and treated with IAXO-101 (**A**). Placental vascular space (**B**) obtained at G19 from pregnant WT mice: non-infected (black open circle), infected (black filled circle), infected treated with IAXO-101 (blue circle), and infected treated with IAXO-101 vehicle (pink square circle). Serum levels of TNF-α measured by cytometric bead arrays (CBA) (**C**), and parasitemia (**D**) of pregnant WT mice infected and treated with IAXO-101 (**A**). As a control, infected pregnant mice were treated with the IAXO-101 vehicle. Mice were infected with 1 × 10^5^ iRBCs i.v. on G13. IAXO-101 treatment was conducted at the same day of the infection with 5 mg/Kg/day by six consecutive days. The animals were euthanized on the 6^th^ day post-treatment. The maternal blood space areas on labyrinth layer were quantified in relation to the total area of the placenta by using an automated morphometric analysis method. Data are presented as scatter plot with an indication of the median. One-way ANOVA: *P-value < 0.05; **P-value < 0.01; ***P-value < 0.001.

Finally, to verify if a TLR4 blockage can revert the phenotype associated with the decreased body weight in fetuses, the infected mice were treated starting on G13 for six days with the CD14/TLR4 antagonist IAXO-101^21–23^. The fetuses born from mothers treated with IAXO-101 presented an intermediate body weight when compared with both infected and non-infected WT mice (Fig. 6A). Interestingly, the treatment partially reversed the placental malaria effects on the fetuses’ weight. Also, the same pattern of results as that of non-infected WT pregnant mice was observed when we evaluated the reduction of vascular space (Fig. 6B). The analysis of the production of the TNF-α in the serum demonstrated a decrease of TNF-α production in infected pregnant mice treated with IAXO-101 when compared with the non-treated infected group (Fig. 6C). Moreover, the treatment of infected pregnant mice with the vehicle has not effect in the TNF-α production. Notably, the IAXO-101 treatment did not have any influence on the course of infection, as shown by the parasitemia levels (Fig. 6D).

## Discussion

Herein we show that activation of the innate immune system, via TLR4, by *P. berghei* NK65 leads to development of experimental placental malaria (PM). Our data shown that TLR4 activation is the main pathway involved in the inflammatory process induced by the parasite in the placenta, and correlates with poor outcomes in PM. The infected pregnant WT mice demonstrated an increase in the pro-inflammatory cytokines in the serum, and at the mRNA level in the placenta, in response to the infection with *P. berghei* NK65. These data correlate with the reduced placental vascular space. In contrast to the infected WT mice, data from TLR4^−/−^ infected mice were similar to those found in non-infected controls. These observations suggest that the absence of functional TLR4 in mice seems to lead to a decrease in the local pro-inflammatory reaction, abolishing the deleterious effects of infection on gestation.

The significance of the TLR gene expression in malaria is not yet conclusive. Recently, Sobota and collaborators^24^ proposed the use of TLRs gene expression in humans as a possible candidate biomarker for severe malaria. In that work, the authors used a case-control design study to compare uncomplicated malaria with severe malaria and verified that in both situations there were an increase of the TLRs and Complement System gene expression. Specifically, for TLRs, these authors demonstrated up-regulation of the TLR2, TLR4, and TLR8. In addition to gene expression data, the TLRs polymorphisms are associated with the malaria susceptibility and pathogenesis^24^. In the same way, the TLRs polymorphisms in pregnant women have been correlated with an increase in susceptibility for placental malaria development^17,18,25^.

Histological analysis showed that TLR2, TLR4, and TLR9 were expressed in the placenta from *P. berghei* infected mice. Other meaningful results of this study highlight a clear correlation between the reduced body weight and TLR4 expression. We show that in the absence of the TLR4 molecule, placental inflammation is reduced, and consequently the fetus weight was not affected by the infection. Consistent with this observation, Arce and collaborators, using a systemic infection model, induced by inoculation of LPS from *Campylobacter rectus* in pregnant mice, demonstrated that TLR4 activation causes low birth weight^26^. Notably, the effects of the LPS via TLR4 on abnormal maternal inflammation have been linked to an increased production of the TNF-α and IL-6^27^. Furthermore, we show that the absence of TLR4 suppresses the production of the TNF-α caused by malaria infection.

In a previous study, we showed that MyD88 is involved in the decreased of IL-6 and TNF-α levels^8^. In human infected pregnant women, the levels of soluble TNF receptors are correlated with *P. falciparum* parasitemia^28^. Moreover, the systemic production of TNF-α and IFN-γ had been implicated in spontaneous abortion^29^ and similar results were obtained in murine models^30–32^. In our studies, we did not observe an increase of reabsorption as a result of systemic TNF-α production, which could be explained by the different gestational times of infection used by us and the studies conducted by others^30^. It should be noted that in our protocol, the moment of infection chosen allows the mice pregnancies carry to term^2^.

The expression of TLR2, TLR4, and TLR9 and the reduction in maternal blood space in the labyrinth layer are relevant finding, since other studies have shown that activation of the maternal inflammatory response alone is sufficient to induce placental damage^33^ and pregnancy loss^34^. Also, administration of the TLR4 agonist in pregnant rats leads to an increase in the TNF-α and IL-6 maternal serum concentrations, as well as a placental TNF-α, IL-6 and IL-1β genes up-regulation^35^, all of which encode pro-inflammatory factors. Further, TLR stimulation induces the fetal reabsorption^36^, preeclampsia^37^, and is related to other pregnancy disorders, such as preterm labor, and intrauterine growth restriction^7^. Taken together, our data provide more evidences that placental damage induced by inflammation can affect offspring outcomes, and corroborate our findings that placental malaria inflammation disrupt the maternal-fetal exchanges, leading to the low birth weight observed in fetuses.

In our *in vitro* studies with transfected cells we showed that *P. berghei* NK65 iRBCs could activate TLR2, TLR4, and TLR9, leading to NF-κB activation. In contrast, our *in vivo* data suggest that TLR2 and TLR9 are not crucial for placental inflammation, since the infection in pregnant TLR2^−/−^ and TLR9^−/−^ mice produced similar results to those found in the infected WT group.

Expanding our previous findings^2,8,19^, we identified in this study that stimulation of the TLR4 pathway by the parasite triggers the innate immune system, causing a local production of TNF-α. This process probably culminates in the placental damage observed in infected mice, since TNF-α can induce tissue injury and placental pathology^38^. Moreover, our results suggest that the local TNF-α expression instead of its systemic production correlated with the placental damage. In addition to what was previously reported, our results indicate that TRIF is also playing an important role in mediating the TLR4 activation, as there were no significant differences between the body weight of fetuses from infected and non-infected TRIF^−/−^ mice. It is noteworthy that, accordingly to the presented data, TNF-α induction has been found to be partially dependent on TRIF^39^.

Since the TLR4 pathway proved to be essential for placental pathogenesis, we sought to investigate the effect of a blockage of this receptor. Using a synthetic antagonist of CD14/TLR4, we observed a partial reversion of the phenotype associated with the decreased body weight in fetuses from WT mice. Notably, this effect was not connected with a significant decrease in mice parasitemia.

Together, the results from this study illustrate the importance of a severe local inflammatory response in PM pathogenesis via TLR4 signalling. Remarkably, the TLR4 signalling blockage by IAXO-101 is presented here as a promising candidate for therapeutic interventions with the aim to reduce the malaria infection risks, in both the mother and the fetus.

## Methods

### Mice and parasites

Eight to 10 weeks-old C57BL/6 (WT), TLR2^−/−^, TLR4^−/−^, TLR9^−/−^, MyD88^−/−^, and TRIF/TICAM^−/−^ mice were bred at the animal facility of the Department of Parasitology from the Institute of Biomedical Sciences at University of São Paulo (ICB/USP). Infected red blood cells (iRBCs) for *in vitro* experiments were obtained from WT mice inoculated intraperitoneally (i.p.) with 1 × 10^5^ erythrocytes infected with GFP-labelled *Plasmodium berghei* NK65. The infection was monitored daily, and the parasitemias were monitored by microscopic examination of Giemsa-stained blood smears (Sigma-Aldrich Inc., St. Louis, MO, USA). The procedures were performed in accordance the national guidelines on animal welfare and were authorized by the ethics committee of the ICB/USP under the protocol no. 065fls104book2.

### Pregnancy monitoring and experimental infection

The detection of vaginal plug and the body weight measurement were used combined to determine the gestation time, as previously described^40^. Females (2 or 3) were placed with one male for 24 h and examined for the presence of the vaginal plug. The first day of gestation (G1) was established by detection of vaginal plugs. Pregnancy progression was monitored every day, based on body weight gain, a successful fertilization being confirmed between G10 and G13 when the females exhibited an average 3–4 g weight increase. Abrupt weight loss was interpreted as an indicator of pregnancy complications or interruption. Pregnant mice were intravenously infected (i.v.) on G13 with 1 × 10^5^ iRBCs, and the parasitemia daily assessed as previously reported^2,19^. Consistent with previous reports G13 was chosen as the optimal time point for the infection as it allows to analyse the pathological features of malaria along the infection in both the mother and the developing fetus, the infection at an earlier stage not allowing reaching the pregnancy term^41^. Placentas for histopathological analysis and RNA extraction were collected at G19. Non-pregnant infected females or non-infected pregnant females were used as controls when appropriate.

### Placenta collection

Placentas from the infected and non-infected females were treated in a similar manner. Briefly, immediately after mice death, placentas were divided into halves: one-half was fixed in 1.6% paraformaldehyde with 20% sucrose for further processing, and the other half was collected in RNAlater® Stabilization Solution (Thermo Fisher Scientific) for subsequent RNA extraction. In some cases, a small piece of placenta was collected for transmission electron microscopy.

### Synchronization and enrichment of parasitized erythrocytes

To obtain mature parasite forms (i.e., trophozoites and schizonts), the iRBCs were synchronized as previously described^40^. Briefly, iRBCs were collected from infected WT mice exhibiting 10–20% parasitemia through cardiac puncture and placed in RPMI 1640 culture medium (Gibco, Life Technologies, Carlsbad, CA, USA) supplemented with 25% fetal bovine serum (FBS) (Sigma-Aldrich) and kept *in vitro* at 37 °C for 14 h in an atmosphere containing 5% CO_2_, 85% N_2_, and 10% O_2_. The infected erythrocytes were enriched using a magnetic separation column (Miltenyl Biotec, Bergisch Gladbach, Germany) to generate cell populations consisting of approximately 95% iRBCs assessed by thick blood smear.

### Transmission electron microscopy

For transmission electron microscopy (TEM), samples of the placenta were fixed overnight in 2.5% glutaraldehyde (Merck Millipore, Billerica, MA, EUA), buffered with 0.1 M Sodium Cacodylate buffer (Na(CH_3_)_2_ AsO_2_ • 3H_2_O, pH 7.4), and post-fixed in 1% osmium tetroxide (OsO_4_), (Merck Millipore) for 3 hours. After dehydration in acetone (Sigma-Aldrich), tissues were embedded in Epoxy embedding medium (Sigma-Aldrich) for 48 hours at 70 °C. Ultra-thin sections of 70 nm were stained with 0.5% uranyl acetate and 8% lead citrate and examined using a Tecnai FEI G20 transmission electron microscope (FEI, Hillsboro, Oregon, USA).

### Immunohistochemistry of frozen sections of placenta

Paraformaldehyde-fixed placenta samples were washed in PBS with 15% sucrose overnight, then embedded in Tissue-Tek^®^ O.C.T. Compound (Sakura Finetek USA, Inc, Torrance, California) and frozen in dry ice. For immunohistochemistry staining, cryostat placenta sections (6 μm thick) were placed on microscope slides, rinsed in PBS for 30 minutes and blocked with 1% bovine serum albumin (BSA) (Sigma-Aldrich). To enhance parasite GFP signal, we used rabbit polyclonal anti-GFP antibody (Molecular Probes, Eugene, OR, USA) and goat anti-rabbit antibody conjugated with Alexa488 (Molecular Probes). To identify macrophages/monocytes we used anti-CD11b biotinylated antibodies (BD Biosciences, Franklin Lakes, NJ, USA), followed by incubation with Rhodamin-Avidin D (Vector Laboratories). Nuclei were stained with DAPI (Invitrogen), and coverslips were mounted with VECTASHIELD® Mounting Media (Vector). Stained sections were examined with a Zeiss Axio Imager M2 light microscope.

### Immunohistochemistry of paraffin-embedded placentas

Sections (5 μm) of paraffin-embedded placentas were deparaffinized in xylene and rehydrated in increasing concentrations of alcohol and distilled water. For heat-induced antigenic retrieval, the sections were placed in 10 mM boiling citrate buffer (pH 6.0) in an electric cooker for 30 min and allowed to cool for 15 min at room temperature (RT). Slides were rinsed in running tap water and 3 times changes of phosphate buffered saline (PBS) for 5 min each. Endogenous peroxidase was blocked by incubating the slides with 3 times changes of 3% H_2_O_2_, 20 min each, at RT in the dark. Unspecific binding sites were blocked by incubation of the sections with Protein Block (Spring Bioscience Co, Pleasanton, CA, USA) for 1 hour at RT. The placental tissues were incubated with primary antibodies anti-TLR2 (IMG-526, IMGENEX, San Diego, CA, USA), anti-TLR4 (IMG-579-A, IMGENEX) and anti-TLR9 (Ab-134368, Abcam, Cambridge, UK) overnight at 4 °C. Primary antibody was washed off, and the slides were rinsed 3 times in PBS and then incubated with the polyvalent secondary antibody-HRP conjugated (Spring Bioscience Co). After rinsing in PBS, the reactions were developed by using the substrate-chromogen DAB (Spring Bioscience Co.). All incubations were performed in a humidity chamber. Placental samples from infected and uninfected control mice were processed simultaneously. The nuclei were counterstained with hematoxylin, and the slides were dehydrated in a series of ethanol dilutions and washed in xylene before putting on coverslips with Tissue-Tek Glas Mounting Media (Sakura Finetek). For negative controls, the primary antibody was omitted.

### Image Acquisition and Analysis

Images of sections were obtained at a 200-fold and a 400-fold magnification on a Zeiss Axio Imager M2 microscope with digital camera Axio Cam HRc using the AxioVision software. An immunohistochemistry semi-quantitative score was defined by using intensity, or protein expression level of positively stained cells (absent = 0, faint = 1, moderate = 2, and intense = 3). We considered as “low expression” TLR intensity scores of 0, 1 or 2, and as “high expression” scores of 3. The slides were scored by two independent observers.

### Histological and Morphometric analysis

Paraformaldehyde-fixed placentas were embedded in paraffin, sectioned at 5 μm, and mounted on silane-coated slides. These sections were dewaxed and rehydrated through descending concentrations of alcohol to distilled water, followed by H&E staining, and microscopical analysis using a Axio Imager M2 microscope coupled to a Axio Cam HRc camera (Carl Zeiss NTS GmBH, Oberkochen, BW, Germany). For the histological and morphometric analysis, the slides were coded and examined by two different observers in a blinded fashion.

Morphometric analysis of the placentas were performed as previously described^2^. In short, the vascular space was quantified by analysing the hematoxylin-eosin (H&E)-stained sections of the placenta. For each section, three areas of the intervillous space were randomly selected for the image acquisition (200x magnification) by using a Axio Cam HRc colour camera (connected to a Axio Imager M2 light microscope). The images were analysed with the image processing and analysis program Image J (NIH, Bethesda, MA, USA). Briefly, the images were subjected to an automated light analysis procedure in which noise removal was applied to ensure colour and image quality standardization across the sections and specimens. The images were given a colour threshold to cover the area corresponding to the blood space lumen. The percentage of coverage was calculated as the ratio between the number of pixels covered by the area defined by the threshold and the total number of pixels in the image. The blood vascular area in each placenta was assessed by the analysis of three non-consecutive sections. The reported results correspond to single pregnant females, and they represent the average result from 3–9 placentas.

### Cytokine measurement

The IL-6, TNF-α, and IL-10 production was assessed in the serum samples using cytometric bead arrays. The procedure was performed in accordance with the manufacturer’s instructions (CBA™, BD Biosciences). The serum samples were analysed on a FACS Canto II (BD Biosciences) with the use of the supplied cytometer setup beads and the FCAP™ Array Software V.3.0.1 (BD Biosciences).

### Gene expression by qRT-PCR

The total RNA was extracted from each placenta obtained at G19 (6th post-infection day) by using the RNeasy mini kit (Qiagen) in accordance with the manufacturer’s protocol for animal tissue (Animal Cell 1). Total RNA (1 μg) was converted into cDNA with First Strand cDNA Synthesis Transcriptor kit (Roche, Penzberg, Germany) using random hexamer primers. The IL-6, IL-10 and TNF-α expression was quantified by using Power SYBR Green PCR Master Mix (Applied Biosystems). The following primer sequences were used: *Il6*, 5′-TTC CAT CCA GTT GCC TTC TT-3′ and 5′-CAG AAT TGC CAT TGC ACA AC-3′; *Il10*, 5′-AAG GAC CAG CTG GAC AAC AT-3′ and 5′-TCA TTT CCG ATA AGG CTT GG-3′; *Tnf*, 5′-AAT GGC CTC CCT CTC ATC AGT T-3′ and 5′-CCA CTT GGT GGT TTG CTA CGA-3′; *Gapdh* 5′-AAC TTT GGC ATT GTG GAA GC and 5′-ACA CAT TGG GGG TAG GAA CA. *Cd3e*, 5′-TCT CGG AAG TCG AGG ACA GT-3′ and 5′-ATC AGC AAG CCC AGA GTG AT-3′; *Klrd1*, 5′-TCA CTC GGT GGA GAC TGA TG-3′ and 5′-AGG CAA ACA CAG CAT TCA GA-3′; *Mgl2*, 5′-GGA TCC CAA AAT TCC CAG TT-3′ and 5′-TCC CTC TTC TCC AGT GTG CT-3′; *Ncf2*, 5′-GCA GTG GCC TAC TTC CAG AG-3′ and 5′-CTT CAT GTT GGT TGC CAA TG-3′; and *cd68*, 5′-AGCTGCCTGACAAGGGACACT-3′ and 5′-AGGAGGACCAGGCCAATGAT-3′. To TLR gene quantification, we use TaqMan® probes as following: *tlr2* (Mm00442346_m1), *tlr4* (Mm00445273_m1), *tlr9* (Mm00446193_m1), and hprt (Mm03024075_m1). The gene expression quantification reactions were performed according to the manufacturer’s instructions on Applied Biosystems 7500 Fast Real-Time PCR System. All results were obtained through the comparative ΔΔCT method^42^ after normalization against Ct values from housekeeping transcripts.

### IAXO treatment

To evaluate the effects of CD14/TLR4 antagonist, pregnant C57BL/6 mice were infected with 1 × 10^5^ iRBC i.v. at G13 and treated with 5 mg/Kg/day of IAXO-101 (Adipogen, San Diego, CA, USA) for 6 days (from G13 to G18). At G19 the data were collected and processed as described above. To evaluate the influence of the vehicle used to dilute IAXO-101 (Ethanol: Phosphate-buffered saline, 1:9) we also treated a group of infected pregnant mice with the vehicle alone.

### Statistical analysis

Statistical differences between two groups were analysed by Unpaired *t-*test, and multi-group comparisons were performed using one-way ANOVA or two-way ANOVA test follow Bonferroni post-hoc test by using the GraphPad Prism version 5.0 software (GraphPad Software, San Diego, CA, USA). P values < 0.05 were considered statistically.

### Data availability

All data generated or analysed during this study are included in this published article (and its Supplementary Information files).

## Electronic supplementary material

Supplementary Information
