# Publisher Correction: TLR4-Mediated Placental Pathology and Pregnancy Outcome in Experimental Malaria

**DOI:** 10.1038/s41598-018-21982-x

**Published:** 2018-03-06

**Authors:** Renato Barboza, Flávia Afonso Lima, Aramys Silva Reis, Oscar Javier Murillo, Erika Paula Machado Peixoto, Carla Letícia Bandeira, Wesley Luzetti Fotoran, Luis Roberto Sardinha, Gerhard Wunderlich, Estela Bevilacqua, Maria Regina D’Império Lima, José Maria Alvarez, Fabio Trindade Maranhão Costa, Lígia Antunes Gonçalves, Sabrina Epiphanio, Claudio Romero Farias Marinho

**Affiliations:** 1 Departamento de Ciências Biológicas, Universidade Federal de São Paulo, Diadema, Brazil; 2 Departamento de Parasitologia, Instituto de Ciências Biomédicas, Universidade de São Paulo, Sao Paulo, Brazil; 3 Hospital Israelita Albert Einstein, São Paulo, Brazil; 4 Departamento de Biologia Celular e do Desenvolvimento, Instituto de Ciências Biomédicas, Universidade de São Paulo, São Paulo, Brazil; 5 Departamento de Genética, Evolução e Bioagentes, Instituto de Biologia, Universidade Estadual de Campinas, Campinas, Brazil; 6 Departamento de Imunologia, Instituto de Ciências Biomédicas, Universidade de São Paulo, São Paulo, Brazil; 7 Departamento de Análises Clínicas e Toxicológicas, Faculdade de Ciências Farmacêuticas, Universidade de São Paulo, São Paulo, Brazil

Correction to: *Scientific Reports* 10.1038/s41598-017-08299-x, published online 17 August 2017

The original version of this Article contained a typographical error in the spelling of the author Claudio Romero Farias Marinho, which was incorrectly given as Cláudio Romerso Farias Marinho. This has now been corrected in the PDF and HTML versions of the Article.

