# Supplementary material for: TLR4-Mediated Placental Pathology and Pregnancy Outcome in Experimental Malaria
Source: Sci Rep. 2017 Aug 17;7:8623. doi: 10.1038/s41598-017-08299-x (PMC5561130; doi:10.1038/s41598-017-08299-x)
Supplement: Supplementary file 1 — Supplementary Information [file 41598_2017_8299_MOESM1_ESM.pdf]

TLR4-Mediated Placental Pathology and Pregnancy Outcome in Experimental Malaria

Renato Barboza[1], Flávia Afonso Lima[2], Aramys Silva Reis[2], Oscar Javier Murillo[2], Erika Paula Machado Peixoto[2], Carla Letícia Bandeira[2], Wesley Luzetti Fotoran[2], Luis Roberto Sardinha[3], Gerhard Wunderlich[2], Estela Bevilacqua[4], Maria Regina D'Império Lima[5], José Maria Alvarez[5], Fabio Trindade Maranhão Costa[6], Lígia Antunes Gonçalves[2], Sabrina Epiphanio[7], Cláudio Romero Farias Marinho[2*]

[1]Departamento de Ciências Biológicas, Universidade Federal de São Paulo, Diadema, Brazil.

[2]Departamento de Parasitologia, Instituto de Ciências Biomédicas, Universidade de São Paulo, São Paulo, Brazil.

[3]Instituto Israelita de Ensino e Pesquisa Albert Einstein, São Paulo, Brazil.

[4]Departamento de Biologia Celular e do Desenvolvimento, Instituto de Ciências Biomédicas, Universidade de São Paulo, São Paulo, Brazil.

[5]Departamento de Genética, Evolução e Bioagentes, Instituto de Biologia, Universidade Estadual de Campinas, Campinas, Brazil.

[6]Departamento de Imunologia, Instituto de Ciências Biomédicas, Universidade de São Paulo, São Paulo, Brazil.

[7]Departamento de Análises Clínicas e Toxicológicas, Faculdade de Ciências Farmacêuticas, Universidade de São Paulo, São Paulo, Brazil.

*marinho@usp.br

Supplementary Information

Additional Methods

Plasmids and NF-κB luciferase assay

COS-7 cells were cultured in 96-well plates ($4x10^4$ cells/well) in Dulbecco's Modified Eagle Medium (DMEM) (Gibco, Carlsbad, CA, USA) supplemented with 10% FBS and gentamicin (Sigma-Aldrich). Once optimal confluence was reached (80-90%), the cells were transfected by using Lipofectamine™ 2000 (Invitrogen, Carlsbad, CA, USA), following the manufacturer's protocol. The amounts of plasmid DNA per well used for transfection were: 25 ng of E-selectin-firefly luciferase, 5 ng of β-actin-*Renilla* luciferase reporter construct, and 25 ng of TLR2, TLR4 or TLR9. Together with the TLR2 and TLR4 plasmids, we added 25 ng of plasmids encoding CD14 and CD36. Cells transfected with the TLR4-coding plasmid were also transfected with 25 ng of myeloid differentiation factor-2 (MD-2). The total DNA concentration was normalized by the addition of an empty vector (pcDNA 3). A TLR9 encoding plasmid was kindly provided by Penn Vector Core (University of Pennsylvania School of Medicine, USA and the other plasmids were a gift from Prof. Igor C. Almeida (University of Texas, USA). Six hours after transfection, the cells were washed and incubated in DMEM medium containing 10% FBS, and rested for 20–24 h. Then, the cells were stimulated for more 6 h with the iRBC and subsequently lysed using "Passive Lysis Buffer" (Promega, Coorporation, Madison, WI, USA) for 5 min at 25°C. As positive controls of TLR2, TLR4, and TLR9 ligands we used PAM3CSK4 (InvivoGen, San Diego, CA, USA), LPS from *E. coli* O111:B4 (Sigma-Aldrich) and CpG ODN (InvivoGen), respectively **(*Supplemental Figure S1*)**. Cell lysates were incubated with the firefly luciferase substrate and Renilla luciferase substrate (Promega). The luminescence was measured in a Lumat LB 9507 luminometer (Berthold Technologies GmbH & Co. Bad Wildbad, BW, German), and the relative NF-κB expression was quantified by using the formula (light output by the E-

selectin-firefly luciferase)/(light output by the β-actin-*Renilla* luciferase control). The relative light unit of samples was normalized by using the protein concentration.

Supplementary figures

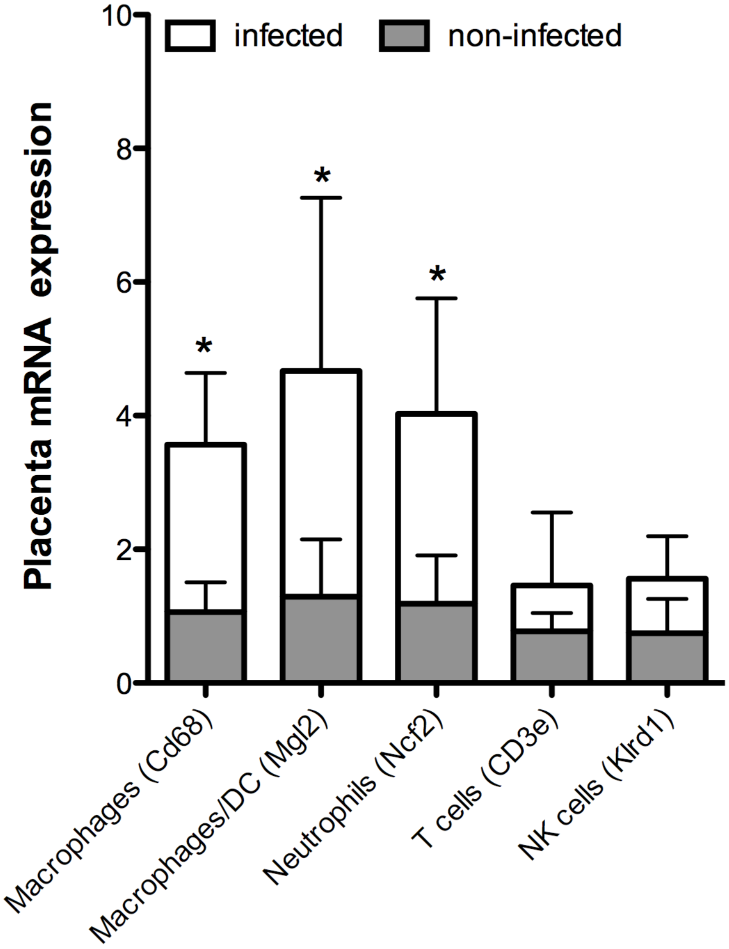

Supplementary Figure 1. Placental gene expression of cell type–specific genes indicating infiltration of inflammatory cells: Cd68 gene for macrophages, Mgl2 for macrophages and dendritic cells (DC), Ncf2 gene for neutrophils, Cd3e gene for T cells, and Klrd1 gene for Natural Killer cells. Data are presented as mean±SD: *P-value<0.05.

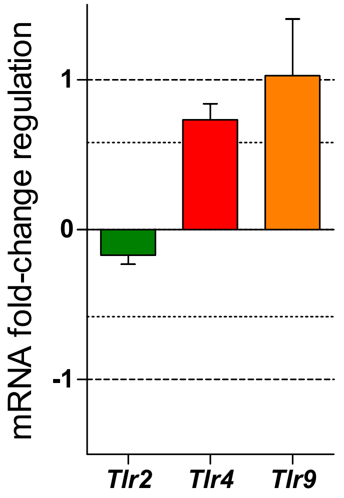

New Supplemental Figure 2. Placental TLR gene expression. Expression of TLR2, TLR4 and TLR9 in placenta from infected mice. Results presented as mRNA expression fold-chance over placental from non-infected mice. Differences in steady state mRNA levels were calculated by comparison with the internal control GAPDH as described.

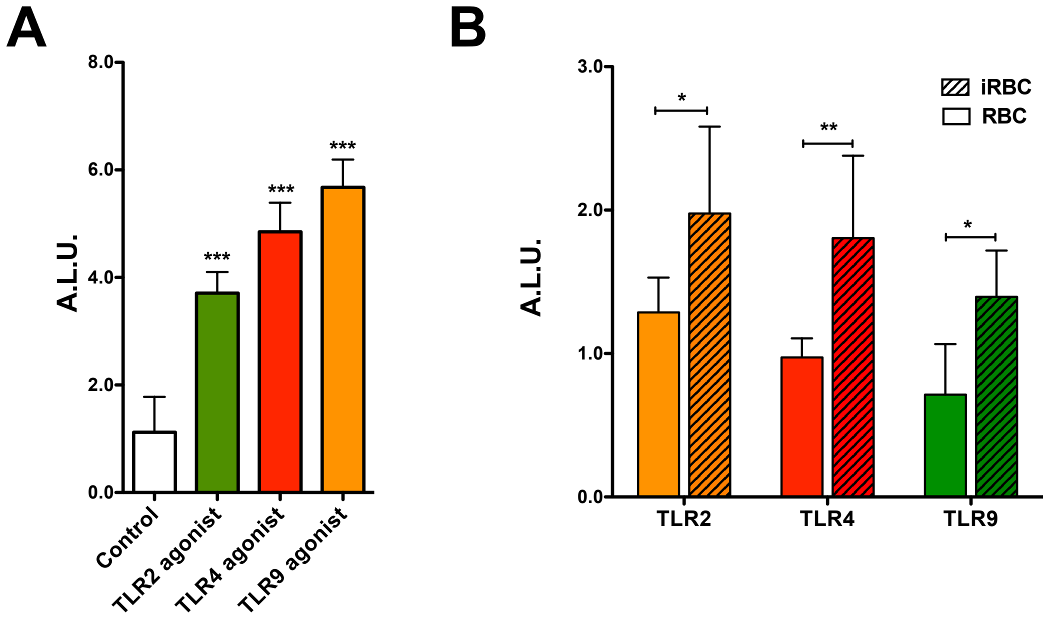

Supplementary Fig. S3. *P. berghei* NK65^{GFP} activate TLR 2, 4, and 9. COS-7 cells were seeded in 96-well plates (4x10^{4} cells/well) and transfected with the TLR plus E-selectin firefly luciferase and β-actin-Renilla luciferase reporter genes. Transfected cells were stimulated with the TLR2 agonist PAM3CSK4 (0.2 μg/ml); the TLR4 agonist LPS (1 μg/ml); or the TLR9 agonist CpG ODN (1 μg/ml) for 6h ***(A)***. The cells were lysed, and the luciferase activity was measured. Data are expressed as arbitrary light units (A.U.L.), calculated by using the formula (light output by the E-selectin firefly luciferase)/light output by the β-actin *Renilla* luciferase control). Transfected cells are stimulated with 3.3x10^{6} *P. berghei* NK65^{GFP} iRBCs ***(B)***. After 6 h, the cells were lysed and the luciferase activity was measured. Data are expressed as fold-induction of E-selectin controlled firefly luciferase. The A.U.L. of samples was normalized using the protein concentration. All data were presented as mean ± SD and they are representative of three independent experiments. One-way ANOVA ***(A)*** and Two-Way ANOVA ***(B)***: **P-value<0.01; ***P-value<0.001.

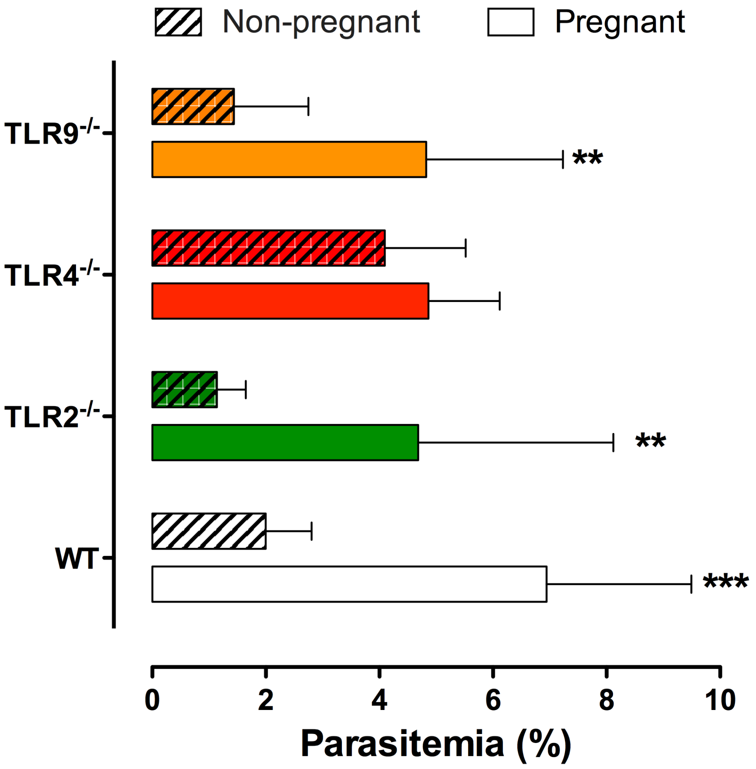

Supplementary Fig. S4: Pregnancy influences the parasitemia levels. Pregnant WT, TLR2$^{-/-}$, TLR4$^{-/-}$, and TLR9$^{-/-}$ mice were intravenously infected on G13 with 1x10^{5} *P. berghei* NK65^{GFP} iRBCs. As a control, non-pregnant mice were infected at the same time. The animals were euthanized at the 6th post-infection day. Data presented as mean ± SD with 10 mice/group. Two-way ANOVA: **P-value<0.01; ***P-value<0.001.

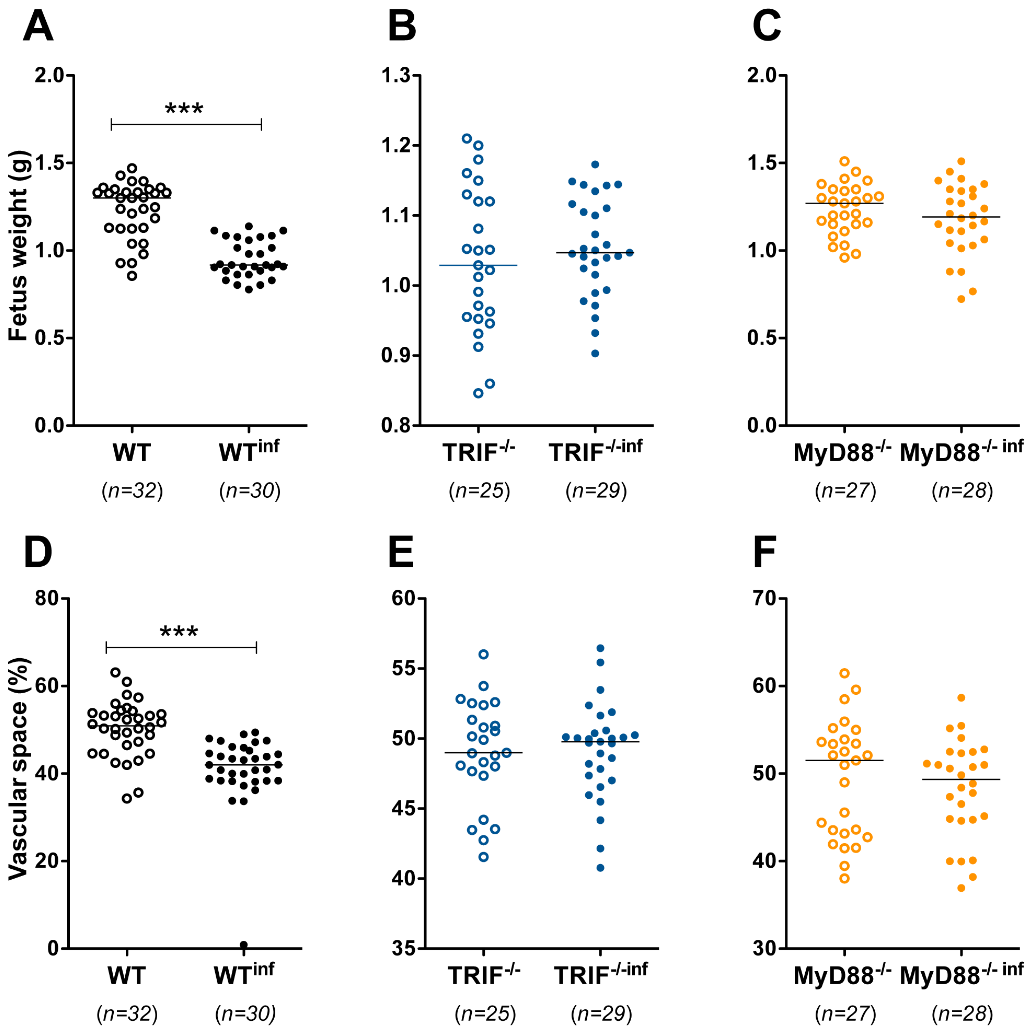

Supplementary Fig. S5. Effect of the malaria infection on fetuses' development from WT, MyD88, and TRIF/TICAM knockout mice. Fetuses weights obtained at G19 from non-infected WT mice (open black circle), infected WT mice (black), non-infected TRIF$^{-/-}$ (open blue circle), infected TRIF$^{-/-}$ mice (blue circle), non-infected MyD88$^{-/-}$ mice (open orange circle) and infected MyD88$^{-/-}$ (orange circle) mice. Mice were infected with $1x10^5$ iRBC i.v. at G13. Data are presented as scatters plot with an indication of the median. Unpaired t-test: ***p<0,0001.
